# Optimisation of lipid-lowering therapy post-Acute Coronary Syndromes: a multidisciplinary, cross-interface novel pharmacy care model within a local cardiac rehabilitation centre

**DOI:** 10.1080/20523211.2025.2523934

**Published:** 2025-07-08

**Authors:** Idil Z. Gul, Sarah Baig, Maria Glover, Mandeep Virdee, Samira Osman, Duncan Jenkins, Zahraa Jalal

**Affiliations:** a School of Pharmacy, School of Health Sciences, College of Medicine and Health, University of Birmingham, Birmingham, UK; b Royal Wolverhampton NHS Trust, Wolverhampton, UK; c Dudley Integrated Health and Care NHS Trust Health and Social Care Centre, Brierley Hill, UK

**Keywords:** Acute coronary syndromes, secondary prevention, lipid-lowering therapies, cardiac rehabilitation, pharmacist-led interventions, non-HDL-C reduction

## Abstract

**Background:**

Acute coronary syndromes (ACS) are a spectrum of diseases that diminish blood flow to the heart and are a major cause of global death. They can be prevented with lipid-lowering therapies (LLTs) but these are often sub-optimally managed in practice. A potential solution could include implementing independent prescribing (IP) pharmacists to optimise LLTs within cardiac rehabilitation services. Aim: To explore the impact of a novel pharmacy service within a cardiac rehabilitation centre (CRC) at a local hospital in the West Midlands UK, on post-ACS patients’ ability to achieve targets of non-HDL-C.

**Methods:**

A retrospective analysis was undertaken at a rehabilitation centre to evaluate pharmacist interventions in lipid management. Inclusion criteria: Post-ACS patients not attaining target levels of non-HDL-C < 2.5 mmol/L, 3 months after discharge. Non-HDL-C levels were also measured 2 months after the pharmacist interventions.

**Results:**

169 post-ACS patients were eligible for treatment. 54% of patients achieved their <2.5 mmol/L of non-HDL-C levels. 25% of these were combination therapy of high-intensity statin and ezetimibe. 36% of patients achieved a 40% reduction of baseline non-HDL levels. 38% did not achieve either target.

**Conclusion:**

This innovative pharmacy role in CR could address the suboptimal LLT management that is common in the post-ACS population. It fosters collaborative care across healthcare sectors, improving patient outcomes and augmenting the probability of reducing ACS risk.

## 1. Background

### 1.1. Sub-optimal lipid-lowering therapies

Acute coronary syndromes (ACS) are a collective term for a group of diseases, encompassing ST, non-ST elevated myocardial infarction (MI) and unstable angina. They are a manifestation of coronary heart disease (CHD) characterised by the disruption of atherosclerotic plaque which induces blockages in the coronary arteries, eliciting severe, acute chest pain (Singh et al., 2021). They result in 80,000 hospital admissions annually in the UK and persist as a predominant global cause of death (Kotecha & Rakhit, 2016; Singh et al., 2021).

In the initial 90–365 days after an ACS event, patients face up to an 8% susceptibility to further complications of cardiovascular disease (CVD) (Bustea et al., 2023; Chi et al., 2022). Therefore, they should receive collaborative healthcare support in addressing modifiable risk factors, such as diabetes and hypertension but particularly hypercholesterolaemia via lipid-lowering therapies (LLTs) (Khan et al., 2023). Despite evidence supporting LLTs’ effectiveness in mortality risk reduction, they are typically underutilised by UK prescribers (Khan & Rakhit, 2022). The National Institute for Health and Care Excellence (NICE) recommend high-intensity statin therapy (HIST), often atorvastatin 80 mg (National Institute for Health and Care Excellence [NICE], 2023a). Patients not achieving a 40% reduction in baseline non-HDL levels after 3 months of HIST initiation may be offered ezetimibe.

Non-attainment of 40% reduction can be a result of suboptimal therapy upon hospital discharge (Khan et al., 2023). 75% receive HIST, and a minute number leave with combination therapy of a HIST with additional LLTs including ezetimibe or PCSK9 inhibitor (PCSK9i) (Khan et al., 2023). PCSK9i, evolocumab, is offered to high-risk patients with LDL-C levels persistently above 4 mmol/L (NICE, 2023a). A likely reason for the lack of additional LLTs in a hospital setting is a demand for quick turnovers which hinders timely lipid optimisation and amplifies CVD risk (NICE, 2022). Combination therapies are significant in this cohort because of their diverse mechanisms of action that aid the increase in the expression of LDL-C receptors and reduction in other atherogenic cholesterols (Chhetry & Jialal, 2021; Masana et al., 2020; NICE, 2023a). These combination therapies reduce non-HDL-C levels more rigorously than monotherapy, therefore more patients are treated to target. It is important to assess non-HDL-C as it comprises of low-density lipoproteins (LDL), triglycerides (TGs), and other atherogenic cholesterols contributing to an augmented CVD risk, offering more comprehensive assessment and management than LDL-C alone (Baker & Forbes, 2010; Guo et al., 2021).

LLT optimisation may also be delayed in the primary care setting due to staff shortages and heightened workload pressure (Desai et al., 2022). This could create a lack of general practice (GP) incentivisation for thorough patient follow-up (Desai et al., 2022). Post-ACS patients followed up by GPs are restricted to a 10-minute review. Consequently, LLT optimisation could be overlooked in this brief encounter (Terry & Mills, 2017). This becomes a pronounced concern in high-risk patients who have high baseline lipid levels who may require the addition of novel therapies mentioned previously. The underutilisation of these evidence-based interventions hampers their potential benefits in reducing CVD risk (Elamin et al., 2019). A lack of GP follow-up consultations can also contribute to ongoing poor adherence (Terry & Mills, 2017). The Global Registry of Acute Coronary Events revealed that patients were most adherent in the first month after ACS (Khan & Rakhit, 2022). After 2 years of the ACS event, only 40% of patients were adherent to their statins which is likely attributed to, but not limited to, apprehensions of statin-associated muscle symptoms (SAMS) (Khatib & Neely, 2022). Although SAMs are experienced by a mere 10% of statin users, a lack of education on the medications’ effects compels 75% of patients in the UK to discontinue within 2 years of initiation (Khatib & Neely, 2022; European Society of Cardiology [ESC], 2022). This attitude contributes to non-attainment of NICE guidelines’ target levels.

### 1.2. Pharmacists in cardiac rehabilitation

A potential solution to sub-optimal LLTs is the integration of an independent prescribing (IP) pharmacist within cardiac rehabilitation services (CRS). Currently, cardiac rehabilitation (CR) is offered to individuals who have experienced an ACS event, have a heart failure diagnosis or have undergone coronary revascularisation (British Heart Foundation [BHF], 2023). In 2022, 85% of ACS patients were referred for CR in the UK (Wilkinson et al., 2019). The high referral rate could be a strategic opportunity in introducing a novel pharmacy service to optimise LLTs. CR programmes are designed to address patients’ emotional, and physical well-being (BHF, 2023). The British Association of Cardiovascular Prevention and Rehabilitation (BACPR) Standards of CR have core components including lifestyle risk factor management, psychosocial health, health education, and medical risk management (British Association for Cardiovascular Prevention and Rehabilitation [BACPR], 2017). They encourage a holistic approach, facilitating the reduction of hospital readmissions and mortality risk, as demonstrated by The Cardiac Rehabilitation Outcome Study (Salzwedel et al., 2020). However, there has been limited exploration of the impact of lipid management within CRS (Salzwedel et al., 2020). A recent small audit conducted in a cardiac rehabilitation centre (CRC) investigated the effectiveness of implementing lipid management pathway (LMP) at a CR centre to optimise LLTs (Jones, 2022). The audit showed a 31% improvement in the number of patients achieving a 50% reduction in baseline LDL-C levels. This audit supports the utilisation of CR as a setting to optimise LLTs.

Randomised controlled trials (RCTs) support the effectiveness of pharmacist-led interventions, including medicines review and education and have shown significance in improving patient clinical outcomes across multiple hospitals and CRCs, thus they could be pivotal in leveraging the benefits of CRS (Ahmed et al., 2022; Casper et al., 2019; Packard et al., 2012).

Our current study seeks to contribute empirical insights into the effectiveness of a prescribing pharmacist-led optimisation of LLTs, utilising a lipid management pathway (LMP), for post-ACS patients within a cardiac rehabilitation setting in a UK hospital.

The objectives of this study are:

1. To determine the percentage of post-ACS patients achieving <2.5 mmol/L of non-HDL-C and/or 40% reduction of baseline non-HDL-C levels, after receiving IP pharmacist interventions, according to evidence-based trials and a NICE-endorsed LMP (Khatib & Neely, 2024; Masana et al., 2020).
2. To evaluate the pharmacist’s application of a stepwise approach to combination therapy of LLTs in line of recommended treatment of post-ACS patients.
3. To evaluate the impact of pharmacist-led optimisation of LLTs in a cardiac rehabilitation setting.

## 2. Methods

### 2.1. Data collection at hospital level

**Figure 1.**
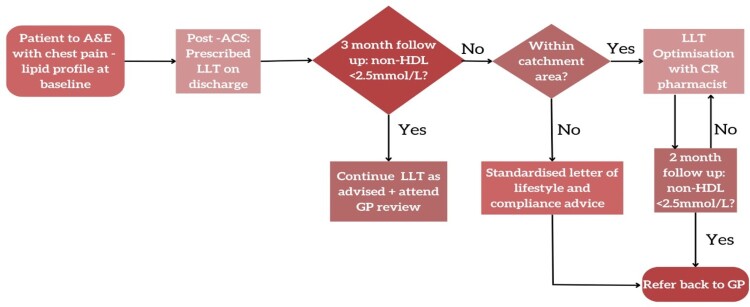
Flow chart providing an overview of a pharmacy service at a cardiac rehabilitation centre to post-ACS patients. A&E = Accident & Emergency. ACS = Acute coronary syndrome. LLT = Lipid lowering therapy. Non-HDL = non-high-density lipoprotein. CR = cardiac rehab. GP = General practitioner.

A retrospective analysis of a novel pharmacy service in CRC took place between November 2019 to October 2023 (Figure 1). At A&E, patients included in the results all had a confirmed diagnosis of ACS. Baseline lipid profiles were recorded, unless this was not available on admission. They were then discharged with LLTs after assessment with the hospital’s CR team and followed up with the local CR team after a 3-month interval. All patients with a presenting complaint of chest pain have a lipid profile on admission. Moreover, Inclusion criteria was based on the 3-month post-event lipid profile rather than on the pre-admission profile.

The Multidisciplinary Team (MDT) at CR included exercise physiologists, cardiologists, nurses, dietitians, pharmacists, and psychologists. Currently, the UK pharmacist's role in CR, in hospital and locally, is confined to educational sessions only including medication education. The new model of care incorporated a pharmacist from secondary care to conduct dedicated medication review, optimisation with telephonic clinics, working 7.5 hours once weekly, focusing on the lipid pathway of care following a cardiac event such as ACS.

### 2.2. Data collection at the local CR clinic

3 months post interventions, patients’ repeat bloods were taken to assess their full lipid profile. The CR pharmacist offered treatment via telephone consultations based on patients’ non-attainment of non-HDL-C < 2.5 mmol/L. Patients beyond the designated catchment area were not eligible for this treatment but received a standardised letter of advice (Figure 1). The cohort achieving target following the 3-month repeat lipid profile were excluded from this study and therefore further analysis of characteristics was not undertaken on this data set.

### 2.3. Pharmacist intervention

Patients received a medication review of LLTs and lifestyle advice including a cardioprotective diet, increased physical activity, and other modifiable factors (NICE, 2023b). Therapeutic decisions were tailored according to the NHS’ NICE-endorsed lipid management pathway (LMP), created to simplify, and encourage LLT optimisation (NICE, 2023a). Novel therapies, inclisiran and PCSK9i, were introduced according to patients’ adherence to NICE-initiated criteria (NICE, 2021). Two months after the interventions, patients received follow-up for reassessments of their lipid profiles. Following achievement of treatment targets, patients were discharged back to their GP care for annual reviews. To ensure collaborative care, the pharmacist ensured liaison by documenting clinical decisions on the patients’ GP records.

### 2.4. Data extraction and analysis

After ethical approval from the university and local hospital, anonymised data of the patients eligible for treatment were reviewed. This included patients’ sex, age, hospital admission date, hospital and pharmacist interventions, and the attained non-HDL-C levels across the timeline. The data extraction was conducted utilising Excel Version 2310. Sex distribution percentages were computed and the average age, along with its standard deviation. The LLTs were identified and categorised based on drug names and doses, facilitated by Excel’s filter tool. The number of patients achieving <2.5 mmol/L of non-HDL-C was quantified and represented in a bar chart. Additionally, a stacked bar chart was created to illustrate the percentage of patients achieving 40% reduction from baseline non-HDL-C levels, the secondary target, before and after pharmacist interventions. These graphical representations serve as visual aids for the analysis of intervention effectiveness.

## 3. Results

### 3.1. Demographics

The mean age of the 169 patients that were eligible for this study was 65.4 ± 11.3 years. The percentage of men and women in the study was 63.9% and 36.1%, respectively (see Table 1).

**Table 1.** Full lipid profile at baseline level of all patients in the CRC. HDL = High density lipoprotein. Non-HDL = non-high-density lipoprotein. N = Total number of patients eligible for the study.

Lipids	Lipid range	No. of patients with following lipid range (N = 169)
	2.4–5.0	90
Total cholesterol (TC) (mmol/L)	5.1–7.0	63
(normal <5.0 mmol/L)	7.1–7.5	5
	7.6–8.5	4
	Not done	7
	0.5–1.1	80
HDL (mmol/L)	1.2–2.0	77
(normal >1 mmol/L for men and 1.2 mmol/L for women)	2.1–4.4	4
	Not done	8
	0–2.4	15
Non-HDL (mmol/L)	2.5–2.0	87
(normal <4.0 mmol/L)	4.1–5.8	54
	6.0–6.8	5
	Not done	8

Table 1 illustrates that 40% of patients’ baseline non-HDL-C levels were above the normal range, < 4 mmol/L, according to national recommendations (NHS, 2022). 3% of patients manifested ‘severe hyperlipidaemia’ with non-HDL-C levels >5.9 mmol/L (NHS, n.d.). 47% of patients’ baseline TC levels were above the normal range, < 5 mmol/L (NHS, 2022).

### 3.2. Pharmacist interventions

**Figure 2.**
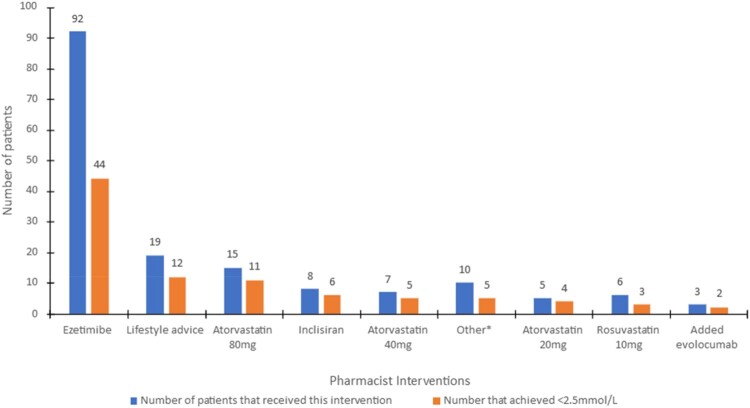
Distribution of pharmacist interventions contributing to the attainment of <2.5 mmol/L of non-HDL. *Antidiabetic medication, intensification to atorvastatin 80 mg with addition of Ezetimibe, compliance advice, rosuvastatin 5 mg & rosuvastatin 40 mg.

In the 2-month follow-up appointment after CR pharmacist interventions, 54% of the cohort achieved the targeted non-HDL-C level (Figure 2). The average non-HDL-C value after the pharmacist’s implementation of the LMP was 2.48 mmol/L ± 0.61. This signifies an augmentation in patients reaching the desired outcome following the intensification of LLTs by the pharmacist.

53% of the cohort was prescribed Ezetimibe 10 mg (whilst on a statin) and 49% of those receiving this intervention achieved the specified target (Figure 2). Amongst the patients who were switched to Atorvastatin 80 mg from another statin or were titrated from a lower dose of atorvastatin, 73% of them achieved target. 4.7% of patients were prescribed Inclisiran, but 75% of those were successful in reaching target. Additionally, despite a small percentage being prescribed evolocumab, 67% of them achieved the target.

The *Other interventions encompass those that contributed 1%, thus their categorisation for analysis purposes. This included *antidiabetic medication, intensification to atorvastatin 80 mg with addition of Ezetimibe, compliance advice, rosuvastatin 5 mg & rosuvastatin 40 mg.

Table 2 shows that in the 3 month follow up, post-hospital discharge interventions, 12% of the cohort achieved a 40% reduction of their baseline non-HDL-C levels three months after the initiation of HIST (atorvastatin 20 mg or 80 mg). Atorvastatin 80 mg yielded a higher success rate, with 10.7% attaining target level, while Atorvastatin 20 mg and patients with no interventions each resulted in 0.6% of patients achieving the target, demonstrating sub-optimal lipid management for most patients from post-discharge interventions.

**Table 2.** Comparison of patients achieving 40% reduction of non-HDL levels * Antidiabetic medication, intensification to atorvastatin 80 mg with addition of Ezetimibe & compliance advice. NA = Not applicable. Non-HDL = non-high-density lipoprotein.

	Patients achieving 40% reduction of baseline non-HDL levels (%)	
Interventions	3 months post-hospital discharge Interventions	2 months post-pharmacist interventions
Atorvastatin 80mg	10.7	2.4
Atorvastatin 20mg	0.60	1.2
No intervention	0.60	0
Addition of Ezetimibe 10mg	NA	21
Inclisiran 284mg	NA	3.6
Lifestyle advice	NA	2.4
Rosuvastatin 10 mg or 40mg	NA	2.4
Added evolocumab	NA	1.8
Other*	NA	1.8

During the 2-month follow-up after the pharmacist interventions, 36.6% of the patients achieved a 40% reduction of their baseline non-HDL-C levels (Table 2). The addition of Ezetimibe resulted in 21% of the patients reaching the target. However, introduction of the novel therapies resulted in 3.6% and 1.8% of patients achieving the target, with monotherapy of Inclisiran and with the addition of evolocumab, respectively. Inclisiran and evolocumab were initiated according to the NHS’ lipid management pathway (LMP) with the average non-HDL-C values they presented with to the pharmacist being 4.21 and 6.1 mmol/L, respectively. The *Other interventions categorise antidiabetic medication, intensification to atorvastatin 80 mg with addition of Ezetimibe & compliance advice, all contributing to 1.8% of the successful interventions.

29% of the cohort achieved targets <2.5 mmol/L and 40% reduction of baseline non-HDL-C levels. 55% of these were on combination therapy of statin and ezetimibe, 4% on combination therapy of statin with evolocumab and 12% were on inclisiran monotherapy. However, 38% of patients did not achieve either target of non-HDL-C. The interventions that did not achieve either target was rosuvastatin 5 mg, atorvastatin 40 mg, and unspecified statin. Furthermore, patients who refused intensification of HIST or discontinued treatment did not achieve either target. Finally, patients with baseline non-HDL-C levels >4.4 mmol/L did not achieve <2.5 mmol/L with statin monotherapy.

## 4. Discussion

### 4.1. Key findings

The incorporation of an IP pharmacist optimising LLTs in CR has yielded a notable increase in the proportion of patients achieving the recommended target lipid levels. The pharmacist took a stepwise approach in introducing additional LLTs to statins. Combination therapy of HIST with ezetimibe was the most prescribed, whilst also demonstrating the highest frequency in achieving both targets. While less commonly prescribed, novel therapies such as inclisiran and evolocumab, exhibited high attainment of <2.5 mmol/L and a 40% reduction of baseline non-HDL-C levels. As a secondary target, a smaller subset of patients achieved a 40% reduction of baseline non-HDL-C levels after pharmacist interventions compared to the interventions post-hospital discharge. Unfortunately, despite the implementation of the NICE-endorsed lipid management pathway (LMP), a third of patients did not achieve either of the predefined targets.

### 4.2. The impact of pharmacists in cardiac rehabilitation

The existing body of literature shows a small number of studies exploring the role of pharmacists within cardiac rehabilitation services (CRS), but they suggest promising effectiveness of their contributions (Ahmed et al., 2022). Contemporary studies depict the current pharmacist’s role in the CR team as patient education, medication adherence, and medicines reconciliation (Casper et al., 2019; Packard et al., 2012). The outcomes resulted in LDL-C reduction, enhanced adherence to secondary prevention medications, and a decrease in drug-related problems (Casper et al., 2019). A cross-sectional audit investigated the impact of pharmacist-initiated interventions at discharge, recommending the prescribing of secondary prevention medicines such as beta blockers and ACE inhibitors (Hassan et al., 2013).

These interventions led to an increase in the utilisation of these therapeutic classes. However, there was a lack of follow-up on the long-term effectiveness of these interventions specifically their impact on hospital readmission rates and mortality (Hassan et al., 2013). A matched cohort study showed that the use of telehealth clinics by a cardiology pharmacist helped to improve adherence of secondary prevention medicines by 13% for patients 12 months post-ACS (Livori et al., 2023). The study suggests that the use of technology to deliver patient care, like in this service evaluation, could be a promising strategy to optimise health outcomes.

While these studies substantiate the benefits of a pharmacist and encourage the incorporation of their interventions within CRS, the current scope of their role does not extend to prescribing LLTs which can limit the supporting findings of this study. Nonetheless, this can encourage further IP pharmacists to take this role to explore the effectiveness of LLTs and other secondary preventive medicines in reducing the recurrence of ACS events in patients.

### 4.3. Combination therapy

#### 4.3.1. Lipid management pathways (LMPs)

In this study, the pharmacist made stepwise prescribing decisions according to NICE-endorsed LMP. Authoritative bodies have varied lipid management recommendations for post-ACS patients (Khan et al., 2023). The European Society of Cardiology (ESC) recommends a 50% reduction in LDL-C levels after 4–6 weeks of HIST initiation, while the UK’s NICE Guidelines suggest a 40% reduction in non-HDL-C levels after 3-months of HIST initiation (Khan et al., 2023). These differences arguably contribute to variations in frequency of target attainment as patients treated by ESC receive LLT optimisation sooner, thus are more likely to achieve reduced LDL-C levels for a longer duration. Although the pharmacist’s initial 3-month follow-up for LLT optimisation was timely, there were delays in the 2-month follow up after pharmacist interventions were introduced. This was a result of delays in patients receiving the required blood test forms and further hindrances in patients attending their repeat blood test appointments. Consequently, patients who may have required further escalation of LLTs were postponed which may contribute to a third of patients not achieving recommended targets. A potential solution is the implementation of digital communication to share necessary forms and send alerts as reminders for scheduled appointments, a practice becoming increasingly popular in primary care. This could facilitate more punctual appointments, encouraging earlier LLT optimisation and attainment of recommended lipid levels.

Additionally, this study placed its primary emphasis on targeting a non-HDL-C level below 2.5 mmol/L. NICE guidelines at the timeframe of pharmacist’s interventions recommended 40% reduction of baseline non-HDL-C levels. Yet RCTs suggest that the more rigorous goal of <2.5 mmol/L of non-HDL-C levels may yield greater reduction of residual risk, particularly for high-risk patients who would otherwise exhibit elevated cholesterol levels even with a 40% reduction in non-HDL-C (Masana et al., 2020). This is further supported by the latest iteration of NICE Guidelines which recommends maintaining non-HDL-C levels at or below 2.6 mmol/L (NICE, 2023b). Moreover, striving for the <2.5 mmol/L threshold may enhance the frequency of target achievement, especially in cases where baseline non-HDL-C levels are unavailable or are already low (NICE, 2022).

#### 4.3.2. Stepwise vs upfront therapy

The pharmacist adopted a stepwise approach in introducing additional LLTs to statins. High-intensity statin therapies (HISTs) are a cornerstone of ACS prevention, with strong evidence demonstrating up to 50% LDL-C reduction (Masana et al., 2020). Although apprehensions of SAMS are a recurrent reason for suboptimal HIST, countless RCTs show ‘<10% of statin users experience side effects caused by the drug’ (ESC, 2022; Krähenbühl et al., 2016).

Therefore, additional LLTs should only be considered if patients experience statin intolerance or are unresponsive to the treatment. Nevertheless, there is debate regarding the appropriateness of statin monotherapy for individuals categorised as ‘very high-risk’ upon discharge, given the likelihood of falling short in achieving the recommended target reduction (Banach et al., 2021; Lewek et al., 2023). Instead of this, The International Lipid Expert Panel advocates the immediate initiation of combination therapy involving a statin and ezetimibe for these patients (Banach et al., 2021). According to this study’s data, this would be beneficial for patients with non-HDL-C levels >4.4 mmol/L as they did not achieve the target <2.5 mmol/L with statin monotherapy. This enhances the probability of attaining target lipid levels as soon as 3-months post-ACS event, thereby averting potential delays and increased CV risks associated with a stepwise approach. The lower the patients’ non-HDL-C levels consistently are early on, the greater reduction in CVD risk, with an RCT reassuring the efficacy and safety of combination therapy’s long-term use (Banach et al., 2021; Giugliano et al., 2017).

For majority of the patients, the IP pharmacist introduced an additional LLT to existing high-intensity statin treatment (HIST). A longitudinal, observational study supports the effectiveness of prescribing HISTs with additional LLTs, particularly ezetimibe and PCSK9i in lowering LDL-C levels compared to HIST monotherapy (Lewek et al., 2023). When HIST is administered in conjunction with ezetimibe, LDL-C reduction can escalate to 60% (Masana et al., 2020). Additionally, the combination of HISTs with PCSK9i has shown an 80% reduction in LDL-C levels within 4 weeks, emphasising the potency of these LLTs. This rigorous reduction is pivotal for high-risk patients experiencing residual risk despite a 50% reduction with HISTs. These regimens facilitate target attainment with a diminished risk of side effects, a prominent concern for statin users (Masana et al., 2020). Note that these studies focus on the reduction of LDL-C levels as it is strongly correlated with diminished morbidity and mortality risk following an ACS event. This study emphasises non-HDL-C reduction as per UK guidelines (Khatib & Neely, 2024; NICE, 2023a).

### 4.4. Why are some patients still not achieving target?

This retrospective study also highlights a subset of patients who failed to achieve the recommended target. This underscores the existing challenges in lipid management including a lack of long-term adherence, particularly when annual follow ups are unlikely, and patient motivation wanes (Desai et al., 2022; Koenig et al., 2023). Consequently, patients’ non-HDL-C levels are not optimally managed, leaving them with continued CVD risk. Additionally, polypharmacy is common in post-ACS patients which can contribute to reduced adherence. The use of less frequently administered LLTs such as PCSK9i could help combat tablet burden but this solution would be limited to those adhering to the NICE initiation criteria, arguably a threshold that is too high (Desai et al., 2022; Elamin et al., 2019). Therefore, other possible resolutions for this may be an extension of the follow-up period with the IP pharmacist to ensure continued monitoring of LLT regimen and patients’ lipid levels being treated to target.

However, a UK real-world study suggests that non-attainment of target lipid levels may stem from some prescribers deviating from the NICE initiation criteria for novel therapies, despite these guidelines being evidence-based, potentially resulting in some high-risk patients receiving suboptimal therapy (Elamin et al., 2019). This can be due to a lack of availability of LLTs such as inclisiran. This was the case in this study, explaining the mere 4.7% of patients being prescribed the LLT. Inclisiran also has a low reported side effect profile, deterring prescribers from its use (Connelly et al., 2023). Moreover, co-morbidities including diabetes, and familial hypercholesterolemia, may contribute to non-attainment of target non-HDL-C levels, despite adherence to guidelines, indicating the complexity of post-ACS management (Burke et al., 2017; Silverio et al., 2023).

### 4.5. Strengths and limitations

The CR pharmacist utilised primary care resources, including GP records to document their interventions and lipid profiles which are accessible to GPs during annual review. This approach is part of the NHS’ Integrated Care Standards which fosters seamless communication across primary and secondary settings, reassuring patients of continued care (National Health Service Education England, 2018). The cohort had a mean age of 65 years which is representative of the wider post-ACS population (Jones, 2022). Additionally, this contemporary model of care is a promising strategy for achieving NHS’ Long-Term Plan which highlights that improving the outcomes of CVD patients is a clinical priority (NHS, n.d.). This was done using the NHS’ lipid pathway, leveraging streamlined management of post-ACS patients (NHS, n.d.).

However, a key limitation is the study’s exclusive reliance on a single CRC which limits its generalisability to other CRCs across the UK. Additionally, the eligibility criteria merely included patients within the catchment area, reducing the study’s external validity. Moreover, there was a lack of access to relevant patient details, such as hospital readmission rates, co-morbidities, ethnicity, and body mass index (BMI). These factors could enable a more comprehensive understanding of the effectiveness of pharmacist interventions as they may influence the attainment of the <2.5 mmol/L or 40% reduction target.

### 4.6. Recommendations

Between 2020 and 2022, there has been a 66% increase in the number of registered IP pharmacists but according to surveys, only 51% claim to prescribe daily (Burns, 2022). Implementing more IP pharmacists into CRS could alleviate workload burden on GPs and encourages the optimisation of LLTs during follow-up appointments. This could be effective in augmenting the number of post-ACS patients attaining target thus a potential reduction in recurrent CVD risk in this cohort (Catapano et al., 2023).

Extending the duration of the pharmacist’s one year follow-up, whether facilitated by the CR pharmacist, community pharmacist, or pharmacy technicians could be significant in encouraging ongoing adherence. Long-term adherence is a growing concern that contributes to the persistence of recurrent ACS events despite effective treatment availability.

Finally, a larger study is recommended to identify a threshold where patients are unlikely to achieve <2.5 mmol/L of non-HDL-C levels with statin monotherapy. According to data collected from this retrospective study, > 4.4 mmol/L is the threshold where <2.5 mmol/L was not achieved, suggesting consideration for upfront therapy of HIST with ezetimibe.

## 5. Future work

The introduction of RCTs researching pharmacist-led LLT interventions could provide quantitative data displaying the statistical significance of these outcomes, facilitating the question of feasibility of this novel service. Feasibility could further be assessed through the incorporation of qualitative studies via interviews that are designed to explore pharmacists’ opinions and concerns about taking on a more clinical role for post-ACS patients in a CR setting. Additionally, it would be recommended that healthcare systems review their current practice and undertake further implementation evaluations like this service evaluation, including economic modelling.

Future service evaluations should have access to patients’ co-morbidities which can contribute to target attainment (Burke et al., 2017). Research shows that patients with co-morbidities are often excluded from RCTs on the secondary management of ACS events, but these people are estimated to make up over 50% of the post-ACS patients (Burke et al., 2017). This could encourage more individualised care for these patients and increase their likelihood of target attainment.

Furthermore, the implementation of longitudinal studies could provide more insight into the impact of non-HDL-C reduction i.e. on hospital admission rates or quality of life. There is a lack of data supporting the correlation between non-HDL-C and risk reduction of ACS events so having more empirical evidence to support this could encourage the consistent measurement of non-HDL-C levels over LDL-C. Currently, both lipid parameters are mentioned throughout NICE guidelines which can spark confusion for HCPs and hesitancy to optimise treatment, further contributing to sub-optimal management (Connolly et al., 2023).

## 6. Conclusion

In conclusion, this evaluation displays a notable improvement in target attainments following the implementation of pharmacist interventions for post-ACS individuals within this cohort. Acknowledging the critical nature of the initial months after an ACS event, incorporating LLT optimisation into CRS could be an advantageous opportunity for post-ACS management. The incorporation of IP pharmacists for this role can facilitate augmented frequency of monitoring, and optimisation of therapies by leveraging the concise NICE-endorsed LMP. This could be particularly profound for high-risk individuals who may require escalation to novel therapies. However, larger, and more thorough evaluations are needed to support the benefits of implementing IP pharmacists into CR.

Overall, this service evaluation indicates that this innovative model may facilitate the augmentation of patients achieving non-HDL-C targets. As a result, there could be a subsequent reduction in hospital admissions, deaths, and morbidities associated with recurrent ACS events, a topical concern in global cardiovascular health.

## References

[CIT0001] Ahmed, A., Guo, P., & Jalal, Z. (2022). A systematic review investigating the role and impact of pharmacist interventions in cardiac rehabilitation. *International Journal of Clinical Pharmacy*, *45*(2), 10.1007/s11096-022-01517-1 PMC10147760 36401764

[CIT0002] BACPR. (2017). The BACPR standards and core components for cardiovascular disease prevention and rehabilitation 2017 (3rd Edition). https://www.bacpr.org/__data/assets/pdf_file/0026/39437/BACPR_Standards_and_Core_Components_2017.pdf.

[CIT0003] Baker, R. A., & Forbes, R. A. (2010). Non-HDL cholesterol. *The Primary Care Companion to the Journal of Clinical Psychiatry*, *12*(5), 10.4088/pcc.09l00940yel PMC3025988 21274366

[CIT0004] Banach, M., Penson, P. E., Vrablik, M., Bunc, M., Dyrbus, K., Fedacko, J., Gaita, D., Gierlotka, M., Jarai, Z., Magda, S. L., Margetic, E., Margoczy, R., Durak-Nalbantic, A., Ostadal, P., Pella, D., Trbusic, M., Udroiu, C. A., Vlachopoulos, C., Vulic, D., & Fras, Z. (2021). Optimal use of lipid-lowering therapy after acute coronary syndromes: A position paper endorsed by the International Lipid Expert Panel (ILEP). *Pharmacological Research*, *166*, 105499. 10.1016/j.phrs.2021.105499 33607265

[CIT0005] British Heart Foundation. (2023). Cardiac rehabilitation. Bhf.org.uk; British Heart Foundation. https://www.bhf.org.uk/informationsupport/support/practical-support/cardiac-rehabilitation.

[CIT0006] Burke, L. A., Rosenfeld, A. G., Daya, M. R., Vuckovic, K. M., Zegre-Hemsey, J. K., Felix Diaz, M., Tosta Daiube Santos, J., Mirzaei, S., & DeVon, H. A. (2017). Impact of comorbidities by age on symptom presentation for suspected acute coronary syndromes in the emergency department. *European Journal of Cardiovascular Nursing*, *16*(6), 511–521. 10.1177/1474515117693891 28198635 PMC5607630

[CIT0007] Burns, C. (2022). Independent prescribing pharmacist numbers total 15,000, increasing by two-thirds in two years. *The Pharmaceutical Journal*. https://pharmaceutical-journal.com/article/news/independent-prescribing-pharmacist-numbers-total-15000-increasing-by-two-thirds-in-two-years.

[CIT0008] Bustea, C., Tit, D. M., Bungau, A. F., Bungau, S. G., Pantea, V. A., Babes, E. E., & Pantea-Roșan, L. R. (2023). Predictors of readmission after the first acute coronary syndrome and the risk of recurrent cardiovascular events – Seven years of patient follow-Up. *Life (chicago, Ill*, *13*(4), 950. 10.3390/life13040950 PMC10140970 37109479

[CIT0009] Casper, E. A., El Wakeel, L. M., Saleh, M. A., & El-Hamamsy, M. H. (2019). Management of pharmacotherapy-related problems in acute coronary syndrome: Role of clinical pharmacist in cardiac rehabilitation unit. *Basic & Clinical Pharmacology & Toxicology*, *125*(1), 10.1111/bcpt.13210 30739389

[CIT0010] Catapano, A. L., Raffaele De Caterina, J. W. J., Klempfner, R., Landmesser, U., Schiele, F., & Sionis, A. (2023). Addressing current challenges in optimization of lipid management following an ACS event: Outcomes of the ACS EuroPath III initiative. *Clinical Cardiology*, *46*(4), 10.1002/clc.23988 PMC10106658 36799113

[CIT0011] Chhetry, M., & Jialal, I. (2021). *Lipid lowering drug therapy*. StatPearls Publishing. https://www.ncbi.nlm.nih.gov/books/NBK541128/. 31082172

[CIT0012] Chi, G., Lee, J. J., Kazmi, S. H. A., Fitzgerald, C., Memar Montazerin, S., Kalayci, A., Korjian, S., Heise, M., Deckelbaum, L. I., Libby, P., Bhatt, D. L., & Gibson, C. M. (2022). Early and late recurrent cardiovascular events among high-risk patients with an acute coronary syndrome: Meta-analysis of phase III studies and implications on trial design. *Clinical Cardiology*, *45*(3), 299–307. 10.1002/clc.23773 35019162 PMC8922536

[CIT0013] Connelly, D., Cohen, D., & McCartney, M. (2023). Fewer than 5,000 people prescribed anticholesterol drug inclisiran in primary care as of July 2023. *The Pharmaceutical Journal*. https://pharmaceutical-journal.com/article/news/fewer-than-5000-people-prescribed-anticholesterol-drug-inclisiran-in-primary-care-as-of-july-2023.

[CIT0014] Connolly, D. L., Zaman, A., Capps, N. C., Bain, S. C., & Fernando, K. (2023). Assessing opinion on lower LDL-cholesterol lowering, and the role of newer lipid-reducing treatment options. *The British Journal of Cardiology*, *30*(69). https://bjcardio.co.uk/2023/05/assessing-opinion-on-lower-ldl-cholesterol-lowering-and-the-role-of-newer-lipid-reducing-treatment-options/. 10.5837/bjc.2023.014 PMC11189157 38911692

[CIT0015] Desai, N. R., Farbaniec, M., & Karalis, D. G. (2022). Nonadherence to lipid-lowering therapy and strategies to improve adherence in patients with atherosclerotic cardiovascular disease. *Clinical Cardiology*, *46*(1), 13–21. 10.1002/clc.23935 36267039 PMC9849440

[CIT0016] Elamin, A., Grafton-Clarke, C., Chen, K., Toba Obafemi, A. L., Katira, R., & Davis, G. (2019). Potential use of PCSK9 inhibitors as a secondary preventative measure for cardiovascular disease following acute coronary syndrome: A UK real-world study. *Postgraduate Medical Journal*, *95*(1120), 61–66. 10.1136/postgradmedj-2018-136171 30709868

[CIT0017] European Society of Cardiology. (2022). Statin intolerance is “over-estimated and over-diagnosed.” https://www.escardio.org/The-ESC/Press-Office/Press-releases/Statin-intolerance-is-over-estimated-and-over-diagnosed#:~:text=Now%2C%20a%20new%20study%20of.

[CIT0018] Giugliano, R. P., Wiviott, S. D., Blazing, M. A., De Ferrari, G. M., Park, J.-G., Murphy, S. A., White, J. A., Tershakovec, A. M., Cannon, C. P., & Braunwald, E. (2017). Long-term safety and efficacy of achieving very Low levels of Low-density lipoprotein cholesterol. *JAMA Cardiology*, *2*(5), 547. 10.1001/jamacardio.2017.0083 28291866 PMC5814987

[CIT0019] Guo, L.-L., Chen, Y., Lin, Q., Tian, F., Xiang, Q.-Y., Zhu, L., Xu, J., Wen, T., & Liu, L. (2021). Non-HDL-C Is more stable than LDL-C in assessing the percent attainment of Non-fasting lipid for coronary heart disease patients. *Frontiers in Cardiovascular Medicine*, *8*, 10.3389/fcvm.2021.649181 PMC8049565 33869310

[CIT0020] Hassan, Y., Kassab, Y., Abd Aziz, N., Akram, H., & Ismail, O. (2013). The impact of pharmacist-initiated interventions in improving acute coronary syndrome secondary prevention pharmacotherapy prescribing upon discharge. *Journal of Clinical Pharmacy and Therapeutics*, *38*(2), 97–100. 10.1111/jcpt.12027 23441979

[CIT0021] Jones, C. (2022). Evaluation of a lipid management pathway within a local cardiac rehabilitation service. *British Journal of Cardiology*, *29*(4), 10.5837/bjc.2022.034 PMC10270301 37332270

[CIT0022] Khan, Z., Gul, A., Yousif, Y., & Gupta, A. (2023). A systematic review of lipid management in secondary prevention and comparison of international lipid management pathways. *Cureus*, *15*(2), 10.7759/cureus.35463 PMC10042622 36994289

[CIT0023] Khan, Z., & Rakhit, R. (2022). Secondary prevention lipid management following ACS: A missed opportunity? *British Journal of Cardiology*, *29*(4), 10.5837/bjc.2022.035 PMC10270297 37332272

[CIT0024] Khatib, R., & Neely, D. (2022). *Statin intolerance pathway*. NHS England. https://www.england.nhs.uk/aac/wp-content/uploads/sites/50/2020/04/statin-intolerance-pathway-v2.pdf.

[CIT0025] Khatib, R., & Neely, D. (2024). *Summary of national guidance for lipid management for primary and secondary prevention of CVD*. NHS England. 10.1001/jamacardio.2020.0013.

[CIT0026] Koenig, W., Lorenz, E. S., Beier, L., & Gouni-Berthold, I. (2023). Retrospective real-world analysis of adherence and persistence to lipid-lowering therapy in Germany. *Clinical Research in Cardiology: Official Journal of the German Cardiac Society*, *113*(6), 10.1007/s00392-023-02257-6 PMC11108924 37603070

[CIT0027] Kotecha, T., & Rakhit, R. D. (2016). Acute coronary syndromes. *Clinical Medicine (London, England)*, *16*(Suppl 6), s43–s48. 10.7861/clinmedicine.16-6-s43 27956440 PMC6329574

[CIT0028] Krähenbühl, S., Pavik-Mezzour, I., & von Eckardstein, A. (2016). Unmet needs in LDL-C lowering: When statins won’t do!. *Drugs*, *76*(12), 1175–1190. 10.1007/s40265-016-0613-0 27456066 PMC4974266

[CIT0029] Lewek, J., Niedziela, J., Desperak, P., Dyrbuś, K., Osadnik, T., Jankowski, P., Witkowski, A., Bielecka-Dąbrowa, A., Dudek, D., Gierlotka, M., Gąsior, M., & Banach, M. (2023). Intensive statin therapy versus upfront combination therapy of statin and ezetimibe in patients With acute coronary syndrome: A propensity score matching analysis based on the PL-ACS data. *Journal of the American Heart Association*, *12*(18), 10.1161/jaha.123.030414 PMC10547305 37671618

[CIT0030] Livori, A., Pol, D., Levkovich, B., & Oqueli, E. (2023). Optimising adherence to secondary prevention medications following acute coronary syndrome utilising telehealth cardiology pharmacist clinics: A matched cohort study. *International Journal of Clinical Pharmacy*, *45*), 10.1007/s11096-023-01562-4 PMC10026199 36940081

[CIT0031] Masana, L., Ibarretxe, D., & Plana, N. (2020). Reasons why combination therapy should be the new standard of care to achieve the LDL-cholesterol targets. *Current Cardiology Reports*, *22*(8), 10.1007/s11886-020-01326-w PMC7305062 32562015

[CIT0032] National Health Service Education England. (2018). Integrated care. Health Education England. https://www.hee.nhs.uk/our-work/integrated-care#:~:text=It%20is%20care%20that%20is.

[CIT0033] NHS. (2022). Cholesterol Levels – High Cholesterol. https://www.nhs.uk/conditions/high-cholesterol/cholesterol-levels/.

[CIT0034] NHS. (n.d.). Accelerated Access Collaborative Lipid Management – Rapid Uptake Product. https://www.england.nhs.uk/aac/what-we-do/innovation-for-healthcare-inequalities-programme/rapid-uptake-products/lipid-management/.

[CIT0035] NICE. (2021). *Inclisiran for treating primary hypercholesterolaemia or mixed dyslipidaemia*. NICE. https://www.nice.org.uk/guidance/ta733/chapter/1-Recommendations. 40802800

[CIT0036] NICE. (2022). *CVD escalation of therapy for secondary prevention scope stakeholder subgroup discussions*. NICE. https://www.nice.org.uk/guidance/ng238/documents/workshop-notes-2.

[CIT0037] NICE. (2023a). *CKS is only available in the UK. NICE*. https://cks.nice.org.uk/topics/lipid-modification-cvd-prevention/management/lipid-therapy-secondary-prevention-of-cvd/.

[CIT0038] NICE. (2023b). *Cardiovascular disease: risk assessment and reduction, including lipid modification* NICE. https://www.nice.org.uk/guidance/ng238/chapter/Recommendations.

[CIT0039] Packard, K., Herink, M., & Kuhlman, P. (2012). Pharmacist’s role in an interdisciplinary cardiac rehabilitation team. *Journal of Allied Health*, *41*(3), 113–117. 117a, 117b. https://pubmed.ncbi.nlm.nih.gov/22968772/. 22968772

[CIT0040] Salzwedel, A., Jensen, K., Rauch, B., Doherty, P., Metzendorf, M.-I., Hackbusch, M., Völler, H., Schmid, J.-P., & Davos, C. H. (2020). Effectiveness of comprehensive cardiac rehabilitation in coronary artery disease patients treated according to contemporary evidence based medicine: Update of the cardiac rehabilitation outcome study (CROS-II). *European Journal of Preventive Cardiology*, *27*(16), 1756–1774. 10.1177/2047487320905719 32089005 PMC7564293

[CIT0041] Silverio, A., Cancro, F. P., Esposito, L., Bellino, M., D’Elia, D., Verdoia, M., Vassallo, M. G., Ciccarelli, M., Vecchione, C., Galasso, G., & De Luca, G. (2023). Secondary cardiovascular prevention after acute coronary syndrome: Emerging risk factors and novel therapeutic targets. *Journal of Clinical Medicine*, *12*(6), 2161–2161. 10.3390/jcm12062161 36983163 PMC10056379

[CIT0042] Singh, A., Museedi, A. S., & Grossman, S. A. (2021). *Acute coronary syndrome*. PubMed; StatPearls Publishing. https://pubmed.ncbi.nlm.nih.gov/29083796/. 29083796

[CIT0043] Terry, M., & Mills, J. (2017). The post-ACS patient: Shared care to improve outcomes. *The British Journal of Cardiology*. https://bjcardio.co.uk/2017/09/the-post-acs-patient-shared-care-to-improve-outcomes/.

[CIT0044] Wilkinson, C., Weston, C., Timmis, A., Quinn, T., Keys, A., & Gale, C. P. (2019). Cohort profile: The myocardial ischaemia national audit project (MINAP). *European Heart Journal – Quality of Care and Clinical Outcomes*, *6*(1), 10.1093/ehjqcco/qcz052 31511861

